# Urachal Adenocarcinoma: A Case Report with Key Imaging Findings and Radiologic-Pathologic Correlation

**DOI:** 10.1155/2018/4935261

**Published:** 2018-02-28

**Authors:** Willian Schmitt, Marta Baptista, Marco Ferreira, António Gomes, Ana Germano

**Affiliations:** ^1^Department of Radiology, Hospital Prof. Doutor Fernando Fonseca, Amadora, Portugal; ^2^Department of Pathology, Hospital Prof. Doutor Fernando Fonseca, Amadora, Portugal; ^3^Department of Surgery, Hospital Prof. Doutor Fernando Fonseca, Amadora, Portugal

## Abstract

Urachal pathologies are rare and can mimic numerous abdominal and pelvic diseases. Differential diagnosis of urachal anomalies can be narrowed down by proper assessment of lesion location, morphology, imaging findings, patient demographics, and clinical history. We report a case of a 60-year-old male, with a history of unintentional weight loss without associated symptoms, who was diagnosed with locally invasive urachal adenocarcinoma. With this article, we pretend to emphasize urachal adenocarcinoma clinical features along with its key imaging findings with radiologic-pathologic correlation.

## 1. Introduction

Urachal cancer (UrC) is an uncommon neoplasm, representing 0.5–2% of all bladder cancer. Although transitional epithelium usually lines the urachus, most urachal tumors are adenocarcinomas (90%) and represent 20%–40% of all primary bladder adenocarcinomas [1]. Despite being such rare entities, the number of publications regarding UrC has increased from 3 (between 1980 and 2005) to 10 cases per year on the last decade [2].

## 2. Case Presentation

We describe the case of a 60-year-old male who presented with a four-month history of 15 kg unintentional weight loss, without associated gastrointestinal or urogenital symptoms. On physical examination a visible, nontender, and nonmobile infraumbilical mass was noted. The patient denied significant past medical or family history. Blood work showed mildly elevated white cell count 14.4 × 10^9^ (normal range: 4–11 × 10^9^) and CRP 9.1 (normal range < 0.30 mg/dL).

**Figure 1:**
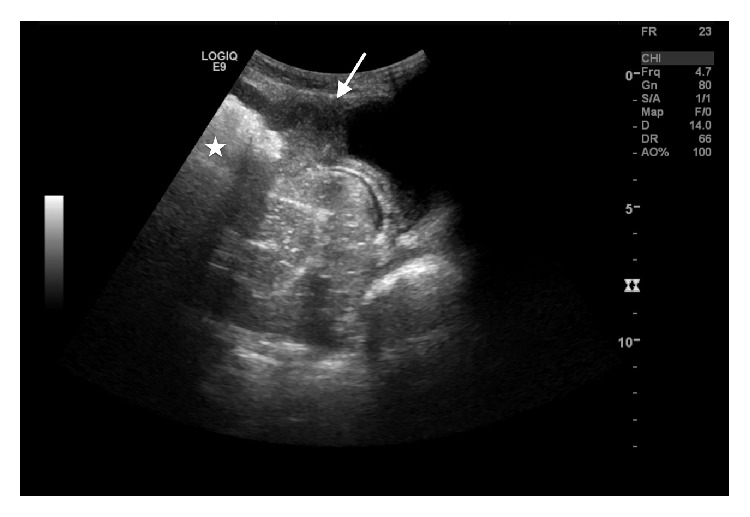
Sagittal US image of the lower abdomen obtained with a convex probe (1–6 Mhz). Large mass filled with gas (star), contacting the superior bladder wall (arrow), with focal thickening. Presence of free abdominal fluid is noted.

An abdominal ultrasound was performed to determine the mass origin. It revealed a midline mass with a central gas-filled cavity contacting the superior bladder wall and extending to the anterior abdominal wall (Figure 1).

**Figure 2:**
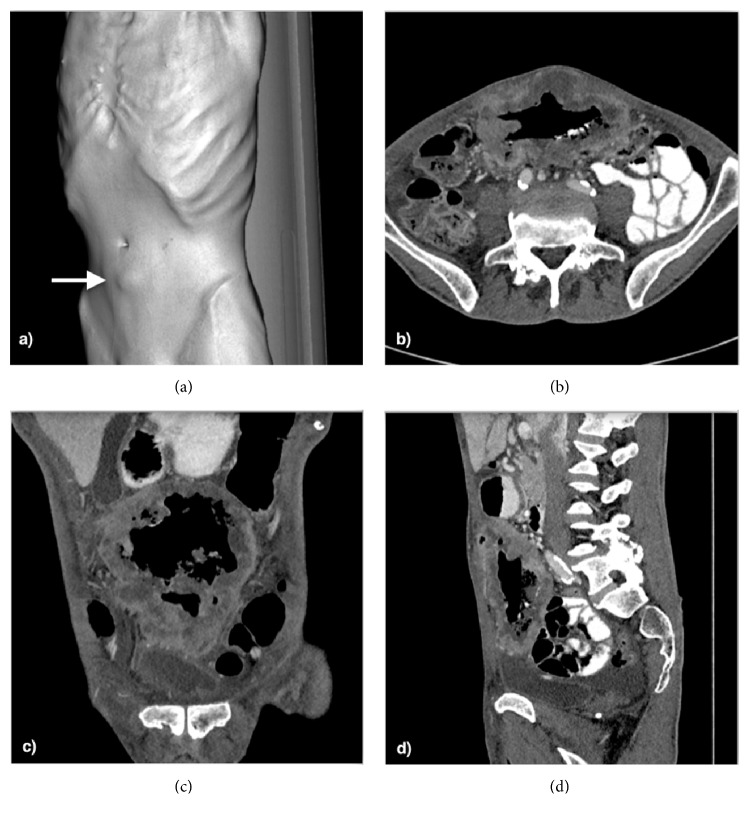
(a) 3D CT reconstruction showing an emaciated patient with a visible infraumbilical mass (arrow). Axial (b), coronal (c), and sagittal (d) contrast-enhanced CT images depicting a large and heterogeneous median abdominal mass, extending from the anterosuperior aspect of the bladder toward the umbilicus.

Additional evaluation with contrast-enhanced abdominopelvic computed tomography (CT) confirmed the presence of a median infraumbilical large intra-abdominal mass, measuring 13 × 8 × 14 cm. Inferiorly it contacted the bladder dome, and anteriorly there was infiltration of the entire thickness of the anterior abdominal wall, invading the umbilicus (Figure 2).

Posteriorly there was no fat interface between the tumor and the transverse colon. Two fistulous tracts were perceived, connecting the mass with this bowel segment. The remaining study was unremarkable, with no sign of distant metastasis.

The morphology of the mass, as well as it aggressive behavior, with abdominal wall, colon, and bladder invasion, suggested a neoplastic lesion. Its location favored a urachal carcinoma as the main diagnosis. Differential diagnosis included a colon carcinoma with abdominal wall and bladder extension, a sarcoma, and an infected urachal remnant.

A colonoscopic study was requested, but it was not completed due to fixation of a colonic loop at 30 cm. After the procedure, discharge of fecal material through skin fistula was noted.

Urgent laparotomy was performed, with total resection of the tumor, with an en bloc resection of the anterior abdominal wall, transverse colon, and superior bladder wall. A temporary colostomy was performed.

**Figure 3:**
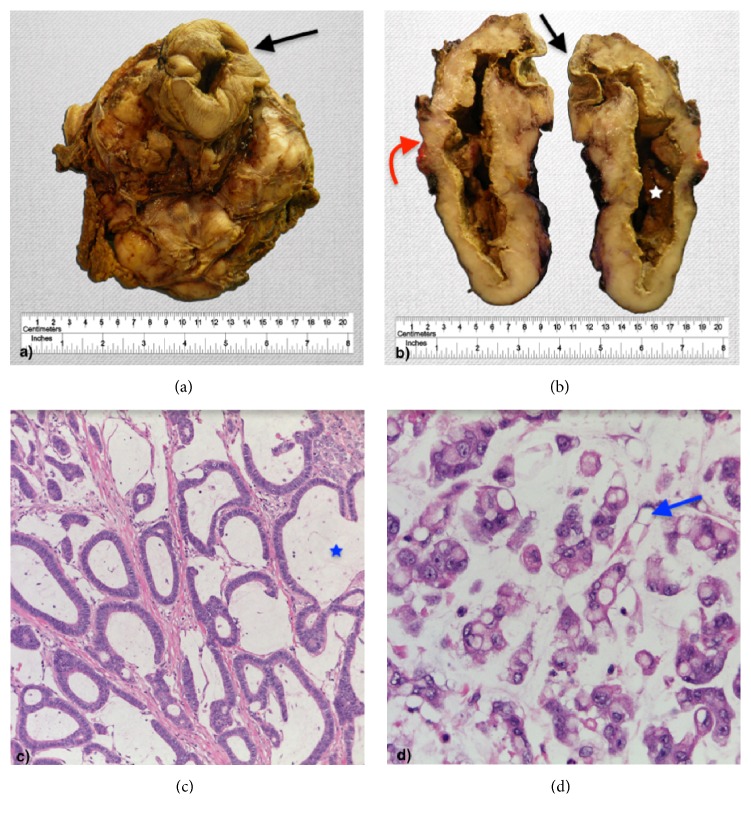
Gross specimen of the tumor ((a) anterior view; (b) cross section), showing a multinodular mass with fistulization to the umbilical skin (black arrows) and involvement of the bladder wall (red arrow) and a central necrotic cavity (star). Histologic image of the resected tumor ((c) H&E 100x; (d) H&E 400x) showing areas with a mucinous pattern (blue star) and a signet ring cell morphology (blue arrow).

Specimen's pathology revealed a poorly differentiated adenocarcinoma with signet ring cell component and mucinous features. It infiltrated the skin, as well as the colonic and bladder walls, with mucosa ulceration (Figure 3).

**Figure 4:**
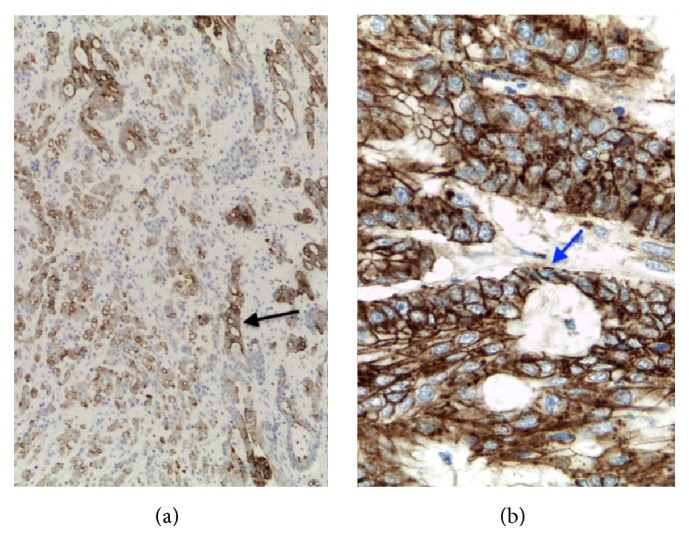
(a) Histologic image of the resected tumor (cytokeratin 34-beta-E12, 100x). Most of the neoplastic cells expressed high molecular-weight cytokeratins (black arrow). (b) Neoplastic cells showing diffuse membranous and cytoplasmic expression of beta-catenin (blue arrow). Note that there is no nuclei staining (beta-catenin, 400x).

Immunohistochemical positivity for 34-beta-E12 and beta-catenin (membrane) was seen (Figure 4), along with focal positivity for CDX2 CK7, and CK20, and uroplakins were nonreactive.

**Figure 5:**
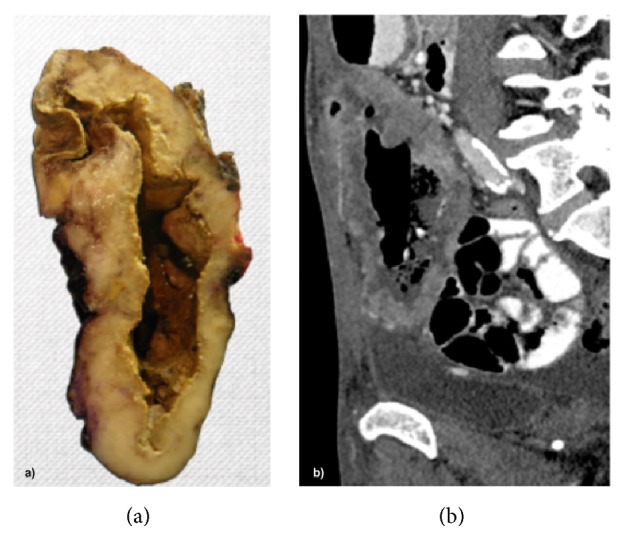
Radiologic-pathologic correlation of the tumor ((a) sagittal CT image; (b) cross section of the gross specimen), showing a large and heterogeneous mass, with thickened wall and central necrotic cavity.

This morphological and immunohistochemical features favored the diagnosis of a urachal adenocarcinoma (Figure 5).

The postoperative period was complicated with surgical wound infection, and the patient was discharged from hospital after 25 days, with wound care instructions and primary care physician referral.

## 3. Discussion

The urachus is an embryological remnant that originates from the involution of the allantois and cloaca, extending from the umbilicus to the bladder dome. During the gestational development, it involutes and becomes the median umbilical ligament, with obliteration of its lumen. Most of the urachus pathologies are found incidentally, and, with the increasing use of cross-sectional imaging, they have become more frequently diagnosed [3].

UrC accounts for 0.01% of all cancers in adults [1]. It was originally described by Hue and Jacquin in 1963 and it has a male predilection, with a mean age of presentation of 60 years (ranging from 40 to 70) [4, 5].

Symptoms do not usually accompany early UrC, which causes patients to present at advanced stages, with local invasion or distant metastases. The most frequently reported symptom is hematuria, followed by abdominal pain, dysuria, and mucosuria [2]. Less frequent clinical presentations include other urogenital related manifestations and nonspecific symptoms such as nausea, diarrhea, or weight loss. In contrast with other bladder cancers, UrC often presents with a palpable lower abdominal mass that along with weight loss was the presentation in our case.

Cystoscopy remains one of the most valuable diagnostic tools for UrC management. It allows a direct visualization of the tumor and it can also determine if the lesion is covered with normal mucosa, as seen in early stages, or if it is a broad-based ulcerated mass [2].

Imaging plays also an invaluable role at UrC workup. On Ultrasound (US), it is commonly recognized as a midline soft tissue mass or a fluid-filled cavity with mixed echogenicity and calcifications. CT scan is often used for local staging and evaluation of distant metastasis. It is usually depicted as a midline mass, superior to the bladder dome and adjacent to the abdominal wall. In the majority of cases, the tumor is mixed solid and cystic, the latter representing its mucin composition. Peripheral calcifications are often seen and are considered pathognomonic for urachal adenocarcinoma. On MRI, focal areas of high signal in T2 indicate the presence of mucinous component. CT and MRI are useful in demonstrating intra- or extravesical extension of the tumor.

The diagnosis of a UrC in biopsy and transurethral resection specimens remains a challenge. The criteria for pathologic diagnosis include the following: (1) location of the tumor in the dome/anterior wall; (2) epicenter of carcinoma in the bladder wall; (3) absence of widespread atypical intestinal metaplasia and cystitis/glandularis beyond the dome/anterior wall; (4) absence of urothelial neoplasia in the bladder; (5) absence of a known primary tumor elsewhere [6, 7].

The most frequent histologic subtype is adenocarcinoma with enteric features with or without mucin production. Some have a signet ring component, as seen in our case, and others have the morphology of colloid carcinomas [5].

Among the used immunomarkers, *β*-catenin and CK7 are the most important in establishing the distinction of urachal from colorectal adenocarcinoma. Diffuse nuclear b-catenin and CK7 help to differentiate urachal adenocarcinoma of enteric subtype (nuclear b-catenin −, CK7 +/−), as seen in our case, from colonic adenocarcinoma (diffuse nuclear b-catenin +, CK7 −) [6].

The main differential diagnosis includes benign urachal tumors, nonurachal carcinomas of the bladder, and metastasis from different organs (prostate, colon, rectum, and female genital tract). Infection of a urachal anomaly can also mimic a UrC, and the diagnosis is often challenging at imaging. In our case, the presence of a midline cavity, anterosuperior to the bladder dome, filled with low attenuation fluid, suggested adenocarcinoma of the urachus as the main diagnosis.

Although several staging systems have been proposed for UrC (Sheldon et al. [8], Ashley et al. [5], and Pinthus et al. [9]), their prognostic relevance has still to be validated in larger series. It is becoming apparent that staging for UrC is more consistent when utterly dichotomized to those confined to urachus, bladder, and perivesical tissue (surgical specimen) and those with spread to the peritoneum and other organs [6, 10]. Mean survival for locally advanced or metastatic disease is 12–24 months, with a 5-year survival rate of 43% [5].

Currently, surgery is the treatment of choice for UrC. The most significant predictor of prognosis is surgical margin status. Because UrC can present with metachronous or synchronous tumors along the urachal tract, the standard surgical approach includes excision of the urachus, umbilicus, and partial/radical cystectomy, combined with pelvic lymphadenectomy [5, 8, 11].

The effective role of adjuvant or neoadjuvant chemotherapy for UrC is not yet established [1, 12]. The fact that most of the available data results from case reports with several chemotherapy regimens and low case numbers does not provide the required statistical significance. Available data suggest that 5-fluorouracil (FU) based chemotherapies are superior to cisplatin-based regimens regarding radiologic tumor response, whereas the combination of 5-FU with cisplatin provides the most beneficial response in metastatic UrC [2, 6]. A fairly recent study described the combination of cytoreductive surgery and hyperthermic intraperitoneal chemotherapy (HIPEC) to be effective in prolonging survival in UrC patients with peritoneal metastases [13].

## Abbreviations

UrC: Urachal cancer
US: Ultrasound
CT: Computed tomography
MRI: Magnetic resonance imaging
FU: Fluorouracil
HIPEC: Hyperthermic intraperitoneal chemotherapy.

## Additional Points

*Key Message*. UrC is a rare malignant tumor and constitutes a clinical challenge. Its diagnosis, management, and treatment decisions rely on a multidisciplinary approach.
